# Chronic hyperglycemia increases the risk of lateral epicondylitis: the Locomotive Syndrome and Health Outcome in Aizu Cohort Study (LOHAS)

**DOI:** 10.1186/s40064-015-1204-3

**Published:** 2015-08-11

**Authors:** Kenichi Otoshi, Misa Takegami, Miho Sekiguchi, Yoshihiro Onishi, Shin Yamazaki, Koji Otani, Hiroaki Shishido, Shunichi Fukuhara, Shinichi Kikuchi, Shinichi Konno

**Affiliations:** Department of Orthopaedic Surgery, Fukushima Medical University School of Medicine, 1, Hikarigaoka, Fukushima City, Fukushima 960-1295 Japan; Department of Preventive Medicine and Epidemiologic Informatics, National Cerebral and Cardiovascular Center, 5-7-1, Fujishirodai, Suita City, Osaka 565-8565 Japan; Institute for Health Outcomes and Process Evaluation Research (iHope International), 513, Nijosagaruakinonomachi, Karasumadori, Chukyoku, Kyoto City, Kyoto 604-084 Japan; Department of Healthcare Epidemiology, Graduate School of Medicine and Public Health, Kyoto University, 54 Shogoinkawahara-cho, Sakyo-ku, Kyoto, Kyoto 606-8507 Japan

## Abstract

**Background:**

Although humeral epicondylitis is a common health problem, there have been no reports that describe its prevalence in Japanese general population, and relatively little is known about its etiology and associated risk factors.

**Questions/purposes:**

This study aimed to clarify the prevalence of humeral epicondilitis in Japanese general population, and investigate the associated risk factors using the data from a cross-sectional study of the Locomotive Syndrome and Health Outcome in Aizu Cohort Study (LOHAS).

**Methods:**

A total of 1,777 participants who participated in health checkups conducted at rural area in Japan in 2010 were enrolled. The prevalence of lateral and medial epicondylitis was investigated. Logistic regression models were performed to examine the relationship between lateral epicondylitis and correlated factors such as occupational status, smoking and alcohol preferences, and medical characteristics.

**Results:**

The overall prevalence of lateral and medial epicondylitis was 2.5 % and 0.3 %, respectively. A shortened version of the disabilities of the arm, shoulder and hand (The *Quick*DASH) score was significantly higher in subjects with lateral epicondylitis than in those without (15.0 ± 12.7 vs 8.5 ± 11.1). Subjects with definite chronic hyperglycemia (HbA1c ≥ 6.5) showed a 3.37-times higher risk of lateral epicondylitis than those with favorable glycemic control (HbA1c < 5.5) (95 % confidence interval (CI) 1.16–8.56). Age and sex, as well as occupational status, smoking and alcohol preference, and other metabolic factors were not significantly related to higher risk of lateral epicondylitis.

**Conclusions:**

Lateral epicondylitis influences activities of daily living. Chronic hyperglycemia might be one of the risk factor for lateral epicondylitis.

**Clinical relevance:**

Chronic hyperglycemia is significantly associated with lateral epicondylitis.

## Background

Lateral epicondylitis is one of the most prevalent disorders of the upper extremity, clinically defined by lateral elbow pain around the lateral epicondyle of the humerus that is provoked by resisted use of wrist and finger extensor (Walker-Bone et al. 2012).

Lateral epicondilitis is characterized by microtears, collagen degeneration, and angioblastic proliferation of the common extensor tendon (Johnson et al. 2007; Kraushaar and Nirschl 1999; Nirschl and Ashman 2004), and may affect muscle fiber type composition (Ljung et al. 1999), neural drive (Alizadehkhaiyat et al. 2009), and stiffness of the muscle–tendon complex (Chourasia et al. 2012; Sesto et al. 2006).

There have been several cross-sectional epidemiologic studies to clarify the etiology and associated risk factors about lateral epicondylitis. A number of studies were carried out among selected occupational populations previously, and suggested an increased risk of epicondylitis associated with strenuous manual tasks such as the repetitive movements of arms and hand with extreme non-neutral postures, static load, and vibration from using handheld vibrating tools (Leclerc et al. 2001). In relation to sports activity, Nirschl and Ashman suggested that the incidence of epicondilitis has been reported to be more often in patient age 35 years or older, high sports activity level, demanding activity technique, and inadequate fitness level (Nirschl and Ashman 2004). Several studies have regarded the relative contributions of preferences and metabolic factors to musculoskeletal disorders. Previous smoking history is an important risk factor for the development of lateral epicondilitis as well as rotator cuff tear and distal biceps tendon rupture (Bodin et al. 2012; Safran and Graham 2002; Titchener et al. 2013), and nicotine tartrate has been shown to induce fibroblast degeneration and irregular fibril organization (Duygulu et al. 2006). Heavy alcohol consumption, more than three standard drinks per day, is associated with an increased risk of early age-related muscular degeneration (Chong et al. 2008). Obesity increases weight on the load-bearing tendons and systemic dysmetabolic factors, triggering subclinical persistent inflammation (Abate et al. 2013; Werner et al. 2005). Hypertension is associated with Achilles tendinopathy, and may have diminution of the local microvascularity as its end-organ effect (Holmes and Lin 2006). Hypercholesterolemia induces cholesterol deposition in the tendons (March et al. 1957). In diabetes, advanced glycation end products deteriorate the biologic and mechanical functions of the tendons and ligaments. However, little is currently known about the relative contributions of preferences and metabolic factors to lateral epicondylitis in the general population (Walker-Bone et al. 2004) and there has been no epidemiologic study reporting the prevalence and determinants of lateral epicondylitis in Japan.

The aim of this large epidemiologic study was to clarify the prevalence of humeral epicondilitis in Japanese general population and investigate the associated risk factors using cross-sectional data from the Locomotive Syndrome and Health Outcome in Aizu Cohort Study (LOHAS) (Otani et al. 2012).

## Methods

Locomotive syndrome, which is a new concept proposed by the Japanese Orthopedic Association in 2007, referring to conditions under which elderly individuals have been receiving care services, or high-risk conditions under which they may soon require care services, due to problems with the locomotive systems (Nakamura 2009). As the population of Japan is aging rapidly, the number of elderly who need nursing care is increased sharply which read to heavy financial burden on society.

The LOHAS is an ongoing prospective cohort study that aims to evaluate the risk of cardiovascular disease, quality of life, medical costs, and mortality attributable to locomotive dysfunction in Japanese subjects (Otani et al. 2012). The subjects were community dwelling individuals over 40 years old who were National Health Insurance (NHI) beneficiaries receiving regular health check-ups conducted by local government each year, in Minami-Aizu Town and Tadami in Fukushima prefecture, Japan. Minami-Aizu-Town and Tadami-Town are located in valleys surrounded by mountains in north-eastern Japan, at longitude 139°46′N, latitude 37°12′E; and longitude 139°18′N, latitude 37°21′E, respectively. The weather in the region is cool, with average daily temperatures of 9.7 °C in Minami-Aizu town and 10.6 °C in Tadami Town. These towns are adjoining and have areas of approximately 745 and 886 km^2^, respectively. The total population of Minami-Aizu Town was 35,728 according to the 2010 population census, with 69.4 % of residents over 40 years old and 21.2 % over 75 years old. The total population of Tadami Town was 4,932 with 72.3 % of residents over 40 years old and 25.8 % over 75 years old. The main industry in the region is agriculture and most resident have remained in the two towns for many years, making this community appropriate for a cohort study. Eligibility criteria included persons who had participated in annual health checkups conducted in 2010. The Research Ethics Committee of our institute approved the study protocol, and written informed consent was obtained from all subjects.

### Survey methodology

This study comprised the following three items: self-completed questionnaire, special health checkup, and physical examination of locomotive function. Self-completed questionnaire forms were distributed to the subjects before an annual health checkup and collected on the day of the checkup. Items on the questionnaire included the subject’s sex, age, occupation, pain and disability of the upper extremities, and general health-related quality of life (HRQOL). A special health checkup was performed to measure height, weight, waist circumference, systolic blood pressure (SBP), diastolic blood pressure (DBP), and glycated hemoglobin (HbA1c), as well as serum levels of low-density lipoprotein cholesterol (LDL-C), high-density lipoprotein cholesterol (HDL-C), and triglycerides (TG). In addition, subjects were questioned regarding their personal medical, smoking, and alcohol history. Physical examination was performed by experienced orthopedic surgeons to investigate locomotive functions and disorders of the subjects, including joint range of motion, muscle power, and pain provocation test. To achieve the best possible accuracy, the orthopedic surgeons were trained before performing the examinations.

### Definition of humeral epicondylitis

The diagnosis of epicondylitis was based on symptoms and clinical signs during a standardized physical examination. The diagnostic criteria for lateral epicondylitis were (1) tenderness at the lateral epicondyle of the humerus and (2) lateral elbow pain around the lateral epicondyle of the humerus on resisted extension of the wrist or finger with the elbow extended. The diagnostic criteria for medial epicondylitis were (1) tenderness at the medial epicondyle of the humerus and (2) medial elbow pain around the medial epicondyle of the humerus on resisted flexion of the wrist or on resisted forearm pronation with the elbow extended.

### Occupational status

The subject’s current or most long-lasting former occupation was investigated, and classified into the following four categories: manual, nonmanual, service, and the other. Manual work included agriculture, transportation, and manufacturing. Nonmanual work included office work, administration, and professional. Service work included sales and the service industry. The population of unemployed subjects was defined as no occupation (Otani et al. 2012).

### Smoking and alcohol preference

Smoking status and alcohol consumption were investigated by self-administrated questionnaire. Smoking status was categorized into three groups: current smoker (the subject smoked at the time of the checkup), former smoker (the subject had smoked and did not currently smoke), and never smoker. Alcohol consumption was categorized into 4 groups: consumes alcohol every day, sometimes, rarely, and never.

### Medical characteristics

Body mass index (BMI) was calculated from the height and weight measured during the special health checkup. Overweight was defined as BMI ≥25 kg/m^2^ based on the criteria of the Japan Society for the Study of Obesity (Examination Committee of Criteria for 2002).

Hypertension was defined according to the Japanese Society of Hypertension Guidelines for the Management of Hypertension (Ogihara et al. 2009), and categorized into three types based on SBP and DBP: normal (SBP < 130 mmHg and DBP < 85 mmHg), definite (SBP ≥ 140 mmHg or DBP ≥ 90 mmHg), and borderline.

Hyperlipidemia was defined according to the criteria recommended by the Japan Atherosclerosis Society (Hata et al. 2002). The subject must satisfy at least one of the following three criteria: fasting serum LDL-C level >140 mg/dL, TG level >150 mg/dL, or HDL-C level <40 mg/dL.

Chronic hyperglycemia was defined according to the criteria recommended by the Japan Diabetes Society (2010). It is categorized into three types based on HbA1c: favorable control (HbA1c < 5.5), suspected (HbA1c, 5.6–6.4), and definite (HbA1c ≥ 6.5).

Metabolic syndrome was defined according to the criteria of the Examination Committee of Criteria for Metabolic Syndrome in Japan (2005) as the subject’s waist circumference must be at least 85 cm for men and 90 cm for women. In addition, the subject must satisfy at least two of the following three criteria: TG level ≥150 mg/dL, HDL-C level ≤40 mg/dL, or be receiving lipid-lowering therapy; SBP ≥130 mmHg, DBP ≥85 mmHg, or be receiving antihypertensive therapy; and fasting plasma glucose level ≥110 mg/dL or be receiving antihyperglycemic therapy.

### Upper extremity disability and HRQOL

Upper extremity disability was assessed using the Japanese version of the shortened Disabilities of the Arm, Shoulder and Hand (The *Quick*DASH) questionnaire (Imaeda et al. 2006). HRQOL was assessed using the Medical Outcome Study Short Form 12-Item Health Survey (SF-12) (Fukuhara and Suzukamo 2004). The subject’s responses to the SF-12 questions were used to determine scores for mental component summary (MCS) and physical component summary (PCS). The scales for MCS and PCS were derived from eight different subscales: physical functioning role (physical, bodily pain, general health, vitality) and social functioning role (emotional health, mental health).

### Statistical analysis

Both baseline characteristics of analyzed and excluded subjects and clinicodemographic characteristics according to presence of lateral epicondylitis were compared using the Chi squared test for categorical variables and the *t* test for continuous variables. Logistic regression models were performed to examine the relationship between lateral epicondylitis and correlated factors. All tests of statistical significance were two-tailed. P values of less than 0.05 were considered to indicate statistical significance. All analyses were conducted using JMP version 10.0.2 (SAS Institute Inc, Cary, NC).

## Results

### Subject attributes

**Fig. 1:**
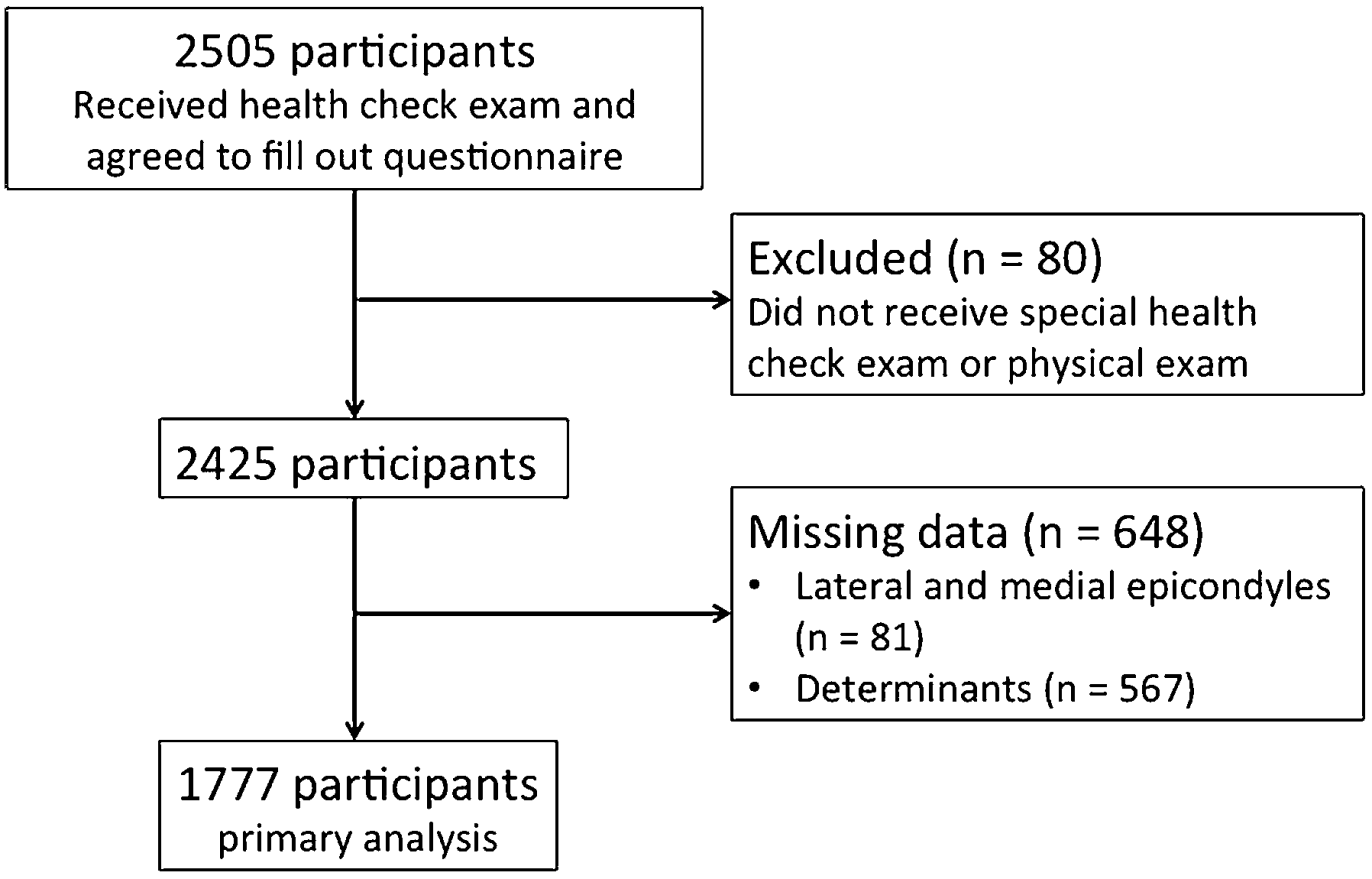
Study flow chart.

Of the 2,505 participants in the LOHAS baseline survey, 728 were excluded for various reasons, leaving 1,777 ultimately enrolled in the present analysis (Fig. 1). Characteristics of the study population are described in Table 1. The subjects included in this analysis were more likely to be younger, male, a smoker, drinker, have a lower QuickDASH score, and have a higher PCS score on the SF-12.

**Table 1:** Baseline characteristics of the analysis population

	All subjects (n = 2,425)	Excluded subjects (n = 648)	Analyzed subjects (n = 1,777)	*P* value
Age (years)				
40–59	370 (15.3)	114 (17.6)	256 (14.4)	<0.001
60–69	847 (34.9)	172 (26.5)	675 (38.0)	
≥70	1,208 (49.8)	362 (55.9)	846 (47.6)	
Sex				
Male	985 (40.6)	211 (32.6)	774 (43.6)	<0.001
Female	1,440 (59.4)	437 (67.4)	1,003 (56.4)	
Occupational categories				
No occupation	1,430 (59.0)	401 (61.9)	1,029 (57.9)	
Nonmanual	134 (5.5)	33 (5.1)	101 (5.7)	0.059
Service	250 (10.3)	63 (9.7)	187 (10.5)	
Manual	426 (17.6)	93 (14.4)	333 (18.7)	
Other	185 (7.6)	58 (9.0)	127 (7.1)	
Smoking status				
Never	1,578 (65.2)	463 (71.9)	1,115 (62.7)	<0.001
Past	587 (24.2)	127 (19.7)	460 (25.9)	
Current	256 (10.6)	54 (8.4)	202 (11.4)	
Alcohol consumption				
Never	770 (32.1)	222 (35.6)	548 (30.8)	<0.001
Every day	559 (23.3)	108 (17.3)	451 (25.4)	
Sometimes	454 (18.9)	122 (19.6)	332 (18.7)	
Rarely	617 (25.7)	171 (27.5)	446 (25.1)	
BMI (kg/m^2^)				
<25	1,134 (63.2)	220 (63.2)	1,124 (63.3)	0.990
≥25	781 (36.8)	128 (36.8)	653 (36.7)	
Past medical history				
Hypertension				
No	1,234 (51.6)	332 (54.0)	902 (50.8)	0.168
Yes	1,158 (48.4)	283 (46.0)	875 (49.2)	
Cerebrovascular disease				
No	2,277 (95.4)	583 (95.6)	1,694 (95.3)	0.804
Yes	110 (4.6)	27 (4.4)	83 (4.7)	
Cardiac disease				
No	2,186 (91.5)	563 (91.8)	1,623 (91.3)	0.697
Yes	204 (8.5)	50 (8.2)	154 (8.7)	
Hyperlipidemia				
No	1,653 (69.3)	433 (71.0)	1,220 (68.7)	0.282
Yes	734 (30.8)	177 (29.0)	557 (31.3)	
Diabetic disease				
No	2,191 (91.9)	564 (92.8)	1,627 (91.6)	0.348
Yes	194 (8.1)	44 (7.2)	150 (8.4)	
Subjective outcome, mean (SD)				
The *Quick*DASH	8.9 (11.4)	9.7 (11.9)	8.7 (11.2)	0.046
SF-12				
PCS	31.9 (12.2)	30.8 (12.1)	32.2 (12.2)	0.030
MCS	45.8 (8.9)	45.9 (8.3)	45.8 (9.1)	0.892

Values represent n (%) of subjects.

*BMI* body mass index, *DASH* disabilities of arm, shoulder and hand, *SF-12* short form 12, *PCS* physical component summary, *MCS* mental component summary, *SD* standard deviation.

### Prevalence of epicondylitis

The prevalences of lateral and medial epicondylitis are described in Table 2. The overall prevalence of lateral and medial epicondylitis was 2.5 % and 0.3 %, respectively. Four subjects had concurrent lateral and medial epicondylitis, with a prevalence of 0.2 %. No significant difference was seen in the prevalence of lateral and medial epicondylitis when comparing men and women and different age groups. There was a trend towards an increased prevalence of medial epicondylitis in men and women between ages 40–59 years. Since the prevalence of medial epicondylitis was too low to analyze statistical determinations, no statistical determinations could be made.

**Table 2:** Prevalence of lateral, medial, and concurrent lateral and medial epicondylitis by age and sex

	Total	Lateral epicondylitis		Medial epicondylitis		Concurrent lateral and medial epicondylitis	
	n	n	Prevalence (%)	n	Prevalence (%)	n	Prevalence (%)
Male							
40–59	113	4	3.5	0	0	0	0
60–69	295	7	2.4	1	0.3	1	0.3
≥70	366	9	2.5	0	0	0	0
All	774	20	2.6	1	0.1	1	0.1
Female							
40–59	143	5	3.5	2	1.4	1	0.7
60–69	380	6	1.6	1	0.3	0	0
≥70	480	13	2.7	2	0.4	2	0.4
All	1,003	24	2.4	5	0.5	3	0.3
Total	1,777	44	2.5	6	0.3	4	0.2

### Clinicodemographic factors in subjects with and without lateral epicondylitis

The frequencies of clinicodemographic factors in subjects with and without lateral epicondylitis are described in Table 3. Only chronic hyperglycemia was associated with lateral epicondilitis whereas there were no significant differences in age or sex between subjects with and without lateral epicondylitis. Smoking and alcohol preference, other medical characteristics, and occupational status also showed no association with lateral epicondylitis. The *Quick*DASH score was significantly higher in those with lateral epicondylitis compared with those without (15.0 ± 12.7 vs 8.5 ± 11.1). In HRQOL, there were no significant differences in PCS or MCS score on the SF-12 between subjects with and without lateral epicondylitis.

**Table 3:** Clinicodemographic variables according to presence of lateral epicondylitis

	Total (n = 1,777)	Subjects with lateral epicondylitis (n = 44)	Subjects without lateral epicondylitis (n = 1,733)	*P* value
Age (years)				
40–59	256 (14.4)	9 (20.5)	247 (14.3)	0.360
60–69	675 (38.0)	13 (29.5)	662 (38.2)	
≥70	846 (47.6)	20 (50.0)	824 (47.5)	
Sex				
Male	774 (43.6)	20 (45.5)	754 (43.5)	0.797
Female	1,003 (56.4)	24 (54.5)	879 (56.5)	
Occupational categories				
No occupation	1,029 (57.9)	23 (52.3)	1,006 (58.1)	0.933
Nonmanual	101 (5.7)	3 (6.8)	98 (5.6)	
Service	187 (10.5)	6 (13.6)	181 (10.4)	
Manual	333 (18.7)	9 (20.5)	324 (18.7)	
Other	127 (7.1)	3 (6.8)	124 (7.2)	
Smoking status				
Never	1,115 (62.7)	26 (59.1)	1,089 (62.8)	0.848
Past	460 (25.9)	13 (29.5)	447 (25.8)	
Current	202 (11.4)	5 (11.4)	197 (11.4)	
Alcohol consumption				
Never	548 (30.8)	16 (36.4)	532 (30.7)	0.542
Every day	451 (25.4)	13 (29.5)	438 (25.3)	
Sometimes	332 (18.7)	5 (11.4)	327 (18.9)	
Rarely	446 (25.1)	10 (22.7)	436 (25.4)	
Obesity				
Normal (BMI <25)	1,124 (63.3)	24 (54.5)	1,100 (63.5)	0.225
Overweight (BMI ≥25)	653 (36.7)	20 (45.5)	633 (36.5)	
Abdominal circumference (cm)				
Normal	1,141 (64.2)	27 (61.4)	1,114 (64.3)	0.690
Abnormal	636 (35.8)	17 (38.6)	619 (35.7)	
Mets				
No	1,458 (82.0)	34 (77.3)	1,424 (82.2)	0.403
Yes	319 (18.0)	10 (22.7)	309 (17.8)	
Hypertension				
Normal	485 (27.3)	12 (27.3)	473 (27.3)	0.923
Borderline	529 (29.8)	12 (27.3)	517 (29.8)	
Definite	763 (42.9)	20 (45.4)	743 (42.9)	
Hyperlipidemia				
Normal	1,188 (66.9)	30 (68.2)	1,158 (66.8)	0.850
Definite	589 (33.1)	14 (31.8)	575 (33.2)	
Chronic hyperglycemia				
Favorable control (HbA1C <5.5)	1,181 (66.5)	23 (52.3)	1,158 (66.8)	0.025
Suspected (5.5 ≤ HbA1C < 6.4)	498 (28.0)	15 (34.1)	483 (27.9)	
Definite (HbA1C ≥6.5)	98 (5.5)	6 (13.6)	92 (5.3)	
Subjective outcome, mean (SD)				
The *Quick*DASH	8.7 (11.3)	15.0 (12.7)	8.5 (11.1)	<0.001
SF-12				
PCS	32.2 (12.2)	29.9 (12.1)	32.3 (12.2)	0.209
MCS	45.8 (9.1)	43.8 (9.5)	45.8 (9.1)	0.144

*BMI* body mass index, *Mets* metabolic syndrome, *SBP* systolic blood pressure, *DBP* diastolic blood pressure, *HDL-C* high density lipoprotein cholesterol, *LDL-C* low density lipoprotein cholesterol, *TG* triglyceride, *HbA1c* hemoglobin A1c, *DASH* disabilities of arm, shoulder and hand, *SF-12* short form 12, *PCS* physical component summary, *MCS* mental component summary, *SD* standard deviation.

In multivariable analysis, after controlling for the effects of other covariates, we found that chronic hyperglycemia had a significant association with lateral epicondylitis (Table 4). Subjects with definite chronic hyperglycemia (HbA1c ≥ 6.5) showed a 3.37 times higher risk of lateral epicondylitis than those with favorable glycemic control (HbA1c < 5.5) (95 % CI 1.16–8.56). The odds ratio for suspected chronic hyperglycemia (HbA1c 5.6–6.4) was also high (1.60 times), but not significantly. Age and sex, as well as occupational status, smoking and alcohol preference, and other medical characteristics showed no significant association with higher risk of lateral epicondylitis.

**Table 4:** Multivariable odds ratios mutually adjusted for determinants of lateral epicondylitis

	Lateral epicondylitis	
	Odds ratio	95 % CI
Age (years)		
40–59	1	
60–69	0.51	0.21–1.31
≥70	0.66	0.28–1.67
Sex		
Male	1	
Female	1.02	0.42–2.55
Occupation		
No	1	
Yes	1.27	0.66–2.45
Smoking status		
Never	1	
Past	1.27	0.50–3.27
Current	1.07	0.31–3.18
Alcohol consumption		
Never	1	
Current	0.71	0.36–1.42
Obesity		
Normal	1	
Overweight	1.41	0.71–2.74
Metabolic syndrome		
Normal	1	
Definite	0.93	0.38–2.15
Hypertension		
Normal	1	
Borderline	0.93	0.40–2.16
Definite	1.04	0.50–2.27
Hyperlipidemia		
Normal	1	
Definite	0.81	0.40–1.55
Chronic hyperglycemia		
Favorable control	1	
Suspected	1.60	0.80–3.10
Definite	3.37*	1.16–8.56

* *P* < 0.05.

## Discussion

According to previous studies, the prevalence of lateral and medial epicondylitis has been reported to be 0.7–23 % (Allander 1974; Descatha et al. 2013; Herquelot et al. 2013; McCormack et al. 1990; Ono et al. 1998; Shiri et al. 2006; Walker-Bone et al. 2004) and 0.2–5.0 % (Ono et al. 1998; Shiri et al. 2006; Verhaar 1994; Viikari-Juntura et al. 1991; Walker-Bone et al. 2012), respectively, and tend to be higher in the working population compared with the general population. In this study, the overall prevalence of lateral and medial epicondylitis was 2.5 % and 0.3 %, respectively, which are comparable with previous epidemiologic studies conducted in the general population.

Regarding individual factors, increasing age has been reported to be possible risk factors for epicondylitits, and the highest prevalence has been reported among subjects aged 40–60 years (Leclerc et al. 2001; McCormack et al. 1990; Ono et al. 1998; Verhaar 1994; Viikari-Juntura et al. 1991). In this study, the prevalence of lateral epicondylitis was higher in subjects aged 40–59 years in both men and women whereas there was no significant difference. Increasing degenerative change of common extensor tendon with advancing age and sustained high physical activity level as their younger age might have an important effect on the development of lateral epicondilitis in middle-aged group (Hamilton 1986).

An association between gender and epicondylitis is still controversial (Leclerc et al. 2001; Ono et al. 1998; Viikari-Juntura et al. 1991; Walker-Bone et al. 2004).

There was no difference in the prevalence of lateral epicondilitis between male and female in this study, while several reports described a higher risk in female sex (Ono et al. 1998; Viikari-Juntura et al. 1991).

Several studies have suggested an increased risk of epicondylitis in association with strenuous manual tasks (Descatha et al. 2003, 2013; Herquelot et al. 2013; Walker-Bone et al. 2003a, b). Repetitive forceful flexing or extending of the elbow and wrist, and twisting, rotating, and screwing motions of the forearm with extreme non-neutral postures have been associated with medial or lateral epicondylitis (Bernard 1997; Descatha et al. 2003, 2013; Haahr and Andersen 2003; Herquelot et al. 2013; Kurppa et al. 1991; Ono et al. 1998; Walker-Bone et al. 2003a, b). According to our results, unexpectedly, there was no significant difference in prevalence of lateral epicondilitis among occupational categories. One of the considerable reasons is that the categories using in this study might not reflect the actual amount of manual tasks involving the upper extremities. In addition, the majority of the residents, including unemployed subjects, would be engaged in agriculture as a family business along with their own occupation. To make a precise assessment of association between the occupation and lateral epicondilitis, detailed evaluation of the actual amount of manual tasks at each occupation would be needed.

Regarding metabolic factors, subjects with chronic hyperglycemia demonstrated a significantly higher risk of lateral epicondylitis compared with those with favorable glycemic control in this study. To the present, several experimental and clinical studies have described the association between diabetes mellitus and tendon degeneration. The animal study using the diabetic rat tendonitis models showed that the number of accumulated neutrophils, macrophages, and proliferative cells was decreased, as was the density of newly formed blood vessels in the paratenon and core of the tendon, and that these alterations eventually perturb tendon healing and remodeling (Chibinou and Frenette 2004). Ahmed et al. also reported that tendon healing in a diabetic rat model was impaired, mainly due to altered expression of collagen and matrix metalloproteinase (MMPs), reflecting decreased degradation of matrix proteins and impaired tissue remodeling (Ahmed et al. 1985). Burner et al. reported that decreased synthesis or sulfation of glycosaminoglycans induced by reduction in prostaglandin (PG) levels may contribute to the tendon pathology observed clinically in diabetes (Burner et al. 2012). In the clinical study, Siddiqui et al. reported that patients with diabetes mellitus had significantly higher pain score, reduced grip strength, and higher recurrence rate after open surgical release for lateral epicondylitis, and concluded that poorer healing and remodeling of the tendon might result in persistence of symptoms or subsequent recurrence after surgery (Siddiqui et al. 2011). While some reports described no relationship between diabetes mellitus and lateral epicondylitis (Titchener et al. 2013), our result and previous studies suggests that it might be appropriate to certify that chronic hyperglycemia could be one of the risk factors for lateral epicondylitis.

There are several limitations when interpreting the results of this study. First, the area surveyed in this study was located in very rural area of Japan with a particularly aged- population. In addition, our study population was not sufficiently large to estimate accurately the prevalence and risk factors of lateral epicondilitis in the Japanese general population. Similar epidemiologic study should be done in urban area to eliminate the regional differences and enhance the reliability of statistical analysis.

Second, there was no information on practicing sports such as tennis and golf in this study. To assess the involvement in sports activity to the epicondilitis would be an issue in the future.

Third, there might be some selection bias in this study. Since subjects voluntarily attended the health checkup, relatively healthy and health-conscious individuals may have participated in this study, and some individuals who make regular hospital visits may not come for annual health checkup.

Last, since our research was a cross-sectional study, it is not possible to assess a causal relationship between chronic hyperglycemia and lateral epicondylitis. A prospective cohort study should be done to clarify the causal relationship between chronic hyperglycemia and lateral epicondylitis.

In conclusion, the prevalences of lateral and medial epicondylitis in Japanese general population were comparable with previous epidemiologic studies conducted in other countries. Chronic hyperglycemia could be one of the risk factors for lateral epicondylitis.
